# Multi-functional benefits from targeted set-aside land in a Danish catchment

**DOI:** 10.1007/s13280-020-01375-z

**Published:** 2020-09-12

**Authors:** Fatemeh Hashemi, Brian Kronvang

**Affiliations:** Department of Bioscience, Aarhus University, Vejlsøvej 25, 8600 Silkeborg, Denmark

**Keywords:** Diffuse water pollution, Multi-functional benefits, Nitrogen load reduction, Targeted land use change

## Abstract

In this study, we explored how a targeted land use change in a Danish catchment (River Odense) may provide multi-functional benefits through nitrogen (N)-load reductions to obtain good ecological quality in Odense estuary, protection of N-vulnerable groundwater aquifers, protection of Natura2000 sites and carbon sequestration. An N-load model linked to GIS thematic layers of known protected areas (Natura2000 sites and N-vulnerable groundwater aquifers) was utilised targeting high N-load areas to locate set-aside land. The achieved multi-functional benefits within the catchment and estuary were assessed and cost–benefit assessment was performed by dividing the total welfare costs of the set-aside by the total multi-functional benefits gained from each strategy. The results show that obtaining multi-functional benefits at the lowest cost requires a targeted shift of set-aside from the traditional hot-spot N-load areas to designated protected areas.

## Introduction

Many aquatic action plans aimed at reducing the level of nitrogen (N)-loads from non-point sources have been implemented in Denmark since the late 1980s (Kronvang et al. 2008) and resulted in an almost 50% reduction of N-leaching from the mid-1980s to 2003 (Naturstyrelsen 2014). Nevertheless, N-leaching is still a major concern for meeting the requirements for good ecological status in groundwater and coastal waters (Kronvang et al. 2005).

The Food and Agriculture Package implemented by the Danish Parliament in 2016 introduced new spatially targeted measures for N-load reductions that include specific N-load reduction targets for all coastal water catchments. The present focus in Denmark for enacting a new policy of differentiated agricultural N-mitigation is based on the spatial variation in groundwater N-reduction (i.e. the redox reaction where nitrate is reduced to N_2_, primarily under anaerobic conditions), a result of geological heterogeneity in the subsurface geology. As N-reduction shows significant small-scale variations depending on the hydrogeological and riverine conditions (Hansen et al. 2014), a spatially differentiated approach with measures targeted towards areas with low natural N-reduction will be more cost-effective than the traditional uniform measures (Olesen et al. 2019; Refsgaard et al. 2019). In addition, the Paris Agreement in 2016 entails a focus on carbon sequestration and with regard to this, Denmark is obliged to meet a 30% CO_2_ reduction target for agricultural emissions by 2030. Furthermore, the EU Habitats Directive and the Rio Agreement require protection of biodiversity.

A spatially targeted strategy may also contribute to the ‘green shift’ in that targeted land use change is a current focal point of Danish policy development of multi-functional land consolidation applying based on the Collective Impact Concept described by Johansen et al. (2018). The Danish Society for Nature Conservation and the Danish Agriculture and Food Council have agreed upon common solutions for nature and agriculture with the aim to allocate sufficient resources for quickly (within a few years) achieving tangible results, either via voluntary agreements on the decommissioning of land or de-intensification of farms in areas of up to 100,000 ha.

Multiple studies have been conducted to elucidate the impact of land use changes of which some have concentrated on hydrology and water quality (e.g. LaBeau et al. 2014), whilst others have investigated how changes in land use may impact water quality improvement and how provision of other ecosystem goods (e.g. biofuel feedstock) may impact biodiversity (Gelfand et al. 2013). Several studies have compared the potential of different land uses to achieve multiple goals ranging from environmental and economic to social metrics for native biodiversity and water quality (Kunkel et al. 2008; Parish et al. 2012; Rankinen et al. 2013). Some have focused on the spatial targeting of land use change to obtain good status of estuaries (Kunkel et al. 2008; Hirt et al. 2012; Vermaat et al. 2012), others considered also variation in detailed groundwater N-reduction (Hansen et al. 2017; Hashemi et al. 2018a, c). However, so far, no studies have considered non-targeted benefits of targeted land use change (i.e. multi-functional benefits including enhanced biodiversity, carbon sequestration and aquifer quality for drinking water) simultaneously. Therefore, in order to provide policy makers with the necessary information for responsible political actions, research should address the possible multi-functional impacts of spatially targeted N-mitigation strategies and regulation at landscape scale whilst considering also the cost imposed on society for implementing such strategies. Although environmental assessment of spatially targeted measures is important regarding the N-loading to the aquatic environments, predicting this in a future land use and management perspective is difficult. Thus, scenario studies as predictive tools have been developed to enable proposition of particular solutions for the future and to explore possible reasons for specific current or past conditions (Hashemi et al. 2016). The size of agricultural areas needed for different combinations of N-mitigation measures to reach different environmental targets in the catchments is also of great importance to society. Because a lower need for taking agricultural lands out of production means lower costs to society (Jacobsen and Hansen 2016).

In this study, we focus on the multi-functional benefits potentially obtained by utilising set-aside as a targeted land use change in a catchment for reducing N-losses with the aim to restore good ecological status of an estuary. Our aims are to quantify the multi-functional benefits of good ecological status in estuary for: (i) restoration of groundwater chemical quality; (ii) protection of nature; (iii) fulfilment of formerly set climate goals in Denmark and their related costs. For this purpose, we investigated the outcomes of different strategies for targeted set-aside using a map-based N-load model in a sub-catchment of Odense Fjord, followed by a cost–benefit assessment performed by dividing the total cost (i.e. the welfare cost of agricultural lands being out of production) by the total multi-functional benefits (relative to a maximum score of 400 points, which is the total sum of benefits related to good ecological status of the estuary, restoration of groundwater chemical quality, protection of nature and fulfilment of formerly set climate goals in Denmark) gained from each strategy.

## Materials and methods

### Study area

**Fig. 1:**
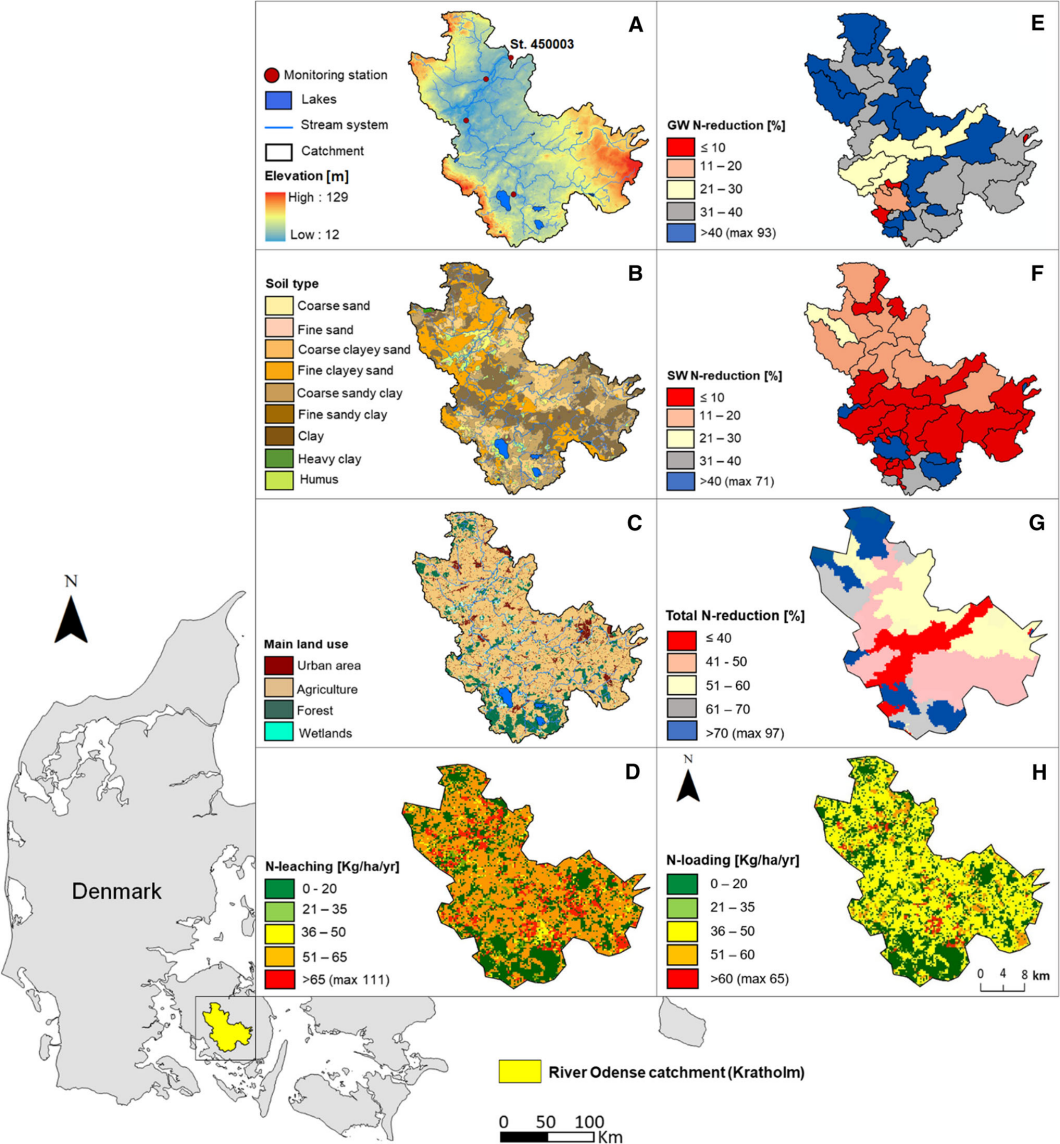
Location of the River Odense catchment (catchment outlet at Kratholm st.450003, south of Odense city) in Denmark. Shown are elevation, the stream system and monitoring stations (**a**), soil type (**b**), main land use (**c**), average N-leaching for the period 1990–2009 at 200 m grid scale (**d**), groundwater N-reduction (GW N-reduction) at sub-catchment scale (**e**), surface water N-reduction (SW N-reduction) at sub-catchment scale (**f**), total N-reduction at sub-catchment scale (**g**), and average N-loading for the period 1990–2009 at 200 m grid scale (**h**)

This study was conducted in an intensively farmed sub-catchment of 486 km^2^ (79%, mainly cropland) of the 1025 km^2^ Odense catchment located on the island of Funen, Denmark (Fig. 1) and covers the area upstream the monitoring station at Kratholm (st.450003). The study area has a temperate and humid climate with a surface geology dominated by loamy-clayey tills. The topographical elevation varies from 12 to 129 m above sea level and observations of daily discharge are available from four stream monitoring stations.

The baseline total N-loading to the Odense Fjord catchment in 2012 was estimated to 1653 tons N yr^−1^, and the 2nd River Basin Management Plans have set a reduction target of 634 tons N yr^−1^ for the area to be achieved 2027 (Danish Nature Agency 2016). The N-loading to the Fjord from the Kratholm outlet studied here in 2012 [normalised to mean climate (discharge)] was 694 tons N yr^−1^ (14.3 kg N ha^−1^) and the N-load reduction target for 2027 is 266 tons N yr^−1^, respectively. The CO_2_ reduction target in the Kratholm outlet is estimated to 37,000 tons CO_2_ equivalents (Danish Energy Agency; Miljø- og Fødevareministeriet 2019). An overview of the location, elevation, soil type, land use, groundwater, surface water and total N-reduction maps at sub-catchment scale and average N-leaching and N-loading maps for the period (1990–2009) at 200 m grid scale is provided in Fig. 1.

### Baseline input data

The annual N-leaching from agricultural land for the period 1990–2009 was calculated using the NLES (version 4) model at grid scale (Fig. 1d). NLES is an empirical regression-based model that estimates N-leaching from information on N-application in fertiliser and manure, cropping sequence, cover crops, soil type and drainage (Kristensen et al. 2008). For each soil type within an agricultural area, yearly N-leaching was calculated using NLES. The soil data used were available from typical Danish soil types, based on top- and sub-soil combinations, where 11–12 typical soil types (see Hashemi et al. 2018c) has been classified for each region (Børgesen et al. 2013). Considering this soil classification, 9 different soil types were available at Odense catchment and 51% of the soil in the catchment is classified as loamy soil and 49% as sandy soils. (Figure 1b). For non-agricultural lands, standard values for N-leaching were used (forest = 5 kg/ha/yr; nature = 2 kg/ha/yr; urban area, surface water = 0 kg/ha/yr). A polygon shape file with yearly N-LES N-leaching was scaled to a 200 m grid for the Odense catchment by calculating an area-weighted average of the N-leaching values for both agricultural and non-agricultural areas within a grid cell (Fig. 1d).

Groundwater N-reduction map originated from the Danish National Nitrogen Model developed by Højberg et al. (2015; Fig. 1e), who used the MIKE SHE model to simulate N-transport and N-reduction for the period of 1990–2010 through particle tracking based on spatially distributed daily N-inputs from N-LES. Next, they converted the annual N-leaching to daily leaching using the DAISY1D model (Abrahamsen and Hansen 2000). N-reduction was simulated assuming instantaneous reduction at the redox interface, defining the transition from oxic to anoxic conditions in the groundwater (Hansen et al. 2014). They also estimated surface water N-reduction (Fig. 1f) as the percent N-reduction of the N-load through surface water within each sub-catchment and for rivers and lakes located on the main stem, and loads from upstream sub-catchments were added to the load generated in each sub-catchment. Next, the percentage of accumulated surface water N-reduction was calculated between a given sub-catchment and the outflow of the downstream sub-catchment in the model setup. Accordingly, the percent of surface water N-reduction was the part removed between a given sub-catchment and the Kratholm (st.450003) monitoring station (Højberg et al. 2015). A detailed description of surface water calculation can be found in a study by Hashemi et al. (2018c).

The total N-reduction map in this study (Fig. 1g) was developed using the following calculation for each sub-catchment within the total catchment:

1 $$R = {\text{GW}} + \left( {\left( {1 - {\text{GW}}} \right) \times {\text{SW}}} \right)$$

where $R$ is the sum of the total N-reduction factors, ${\text{GW}}$ is the N-reduction factor for groundwater (fraction of N-leaching), and ${\text{SW}}$ is the reduction factor for N removed in surface water (fraction of N transported to the surface water).

### N-load calculation using a map-based model

The N-load model is a stationary model with a grid resolution of 200 m; it does not consider variation in the N-fluxes over time and calculates total N-load at the catchment outlet (i.e. N transported out of the catchment at the Kratholm station) after N-reduction processes have removed some of the nitrogen. The N-load (Fig. 1h) was calculated using the N-leaching map at 200 m grid scale and the total N-reduction map at sub-catchment scale as follows:

2 $$N = L \times \left( {1 - R} \right)$$

where *N*, *L* and *R* are N-load, N-leaching and the total N-reduction factor (resulting from Eq. 1), respectively.

### Spatially targeted strategies for reducing the N-load

**Fig. 2:**
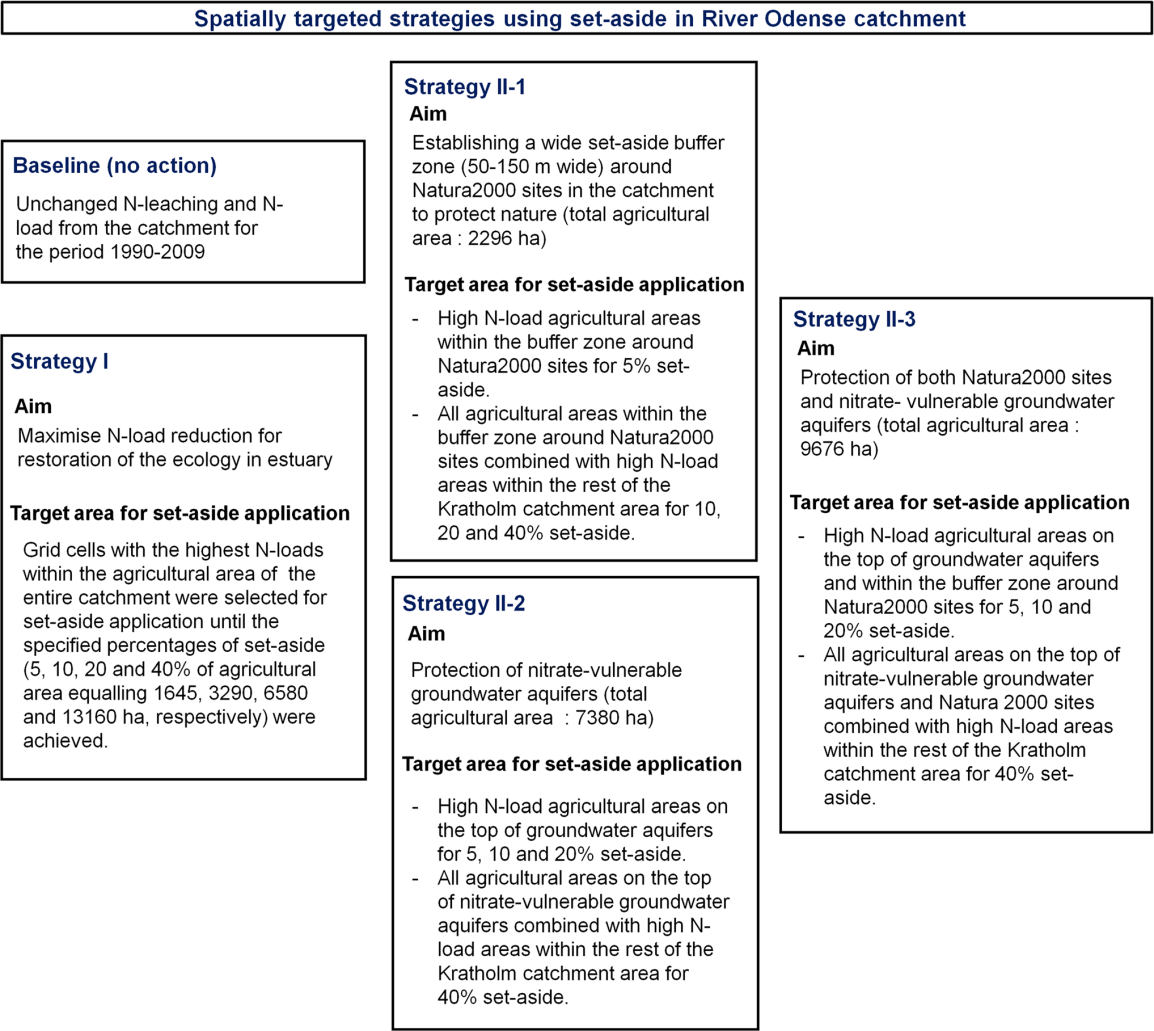
Overview of spatially targeted strategies to obtain multi-functional benefits of set-aside application in the River Odense catchment

Mitigation strategies aimed at achieving the maximum N-load reduction with as large increase in multi-functional benefits as possible, and the strategies were evaluated in terms of the location and area of agricultural lands required for different percentages of set-aside (5, 10, 20 and 40%). Strategies for reducing N-load were implemented according to the scheme presented in Fig. 2 and comprised targeting set-aside: (I) to maximise the N-load reduction for achieving a good ecological status (GES) in Odense estuary and (II) to protect nature and restore groundwater chemical quality and, at the same time, maximise the N-load reduction of the estuary. The baseline (no action) situation was also considered, maintaining the current conditions with no change in average N-leaching and N-load for the period 1990–2009, assuming no changes in regulations.

The spatially targeted strategy in this study was set-aside application in high N-load (hot-spot) areas in different parts of the catchment. The main target for change was the N-leaching input (1990–2009) when planning the strategies that covered only agricultural land in rotation and excluded urban areas, permanent grass, natural vegetation and wetlands. Set-aside is estimated to reduce N-leaching to a fixed value of 12 kg N ha^−1^yr^−1^ (Eriksen et al. 2014).

For strategy (I), grid cells related to the agricultural lands of the entire catchment area with highest N-load contributions were selected as target areas for applying set-aside to reduce N-leaching. This was performed by sequentially applying set-aside to the grid cells with the highest N-loads within agricultural area until the percentages of set-aside were achieved: 5, 10, 20 and 40% equalling 1645, 3290, 6580 and 13160 ha, respectively.

**Fig. 3:**
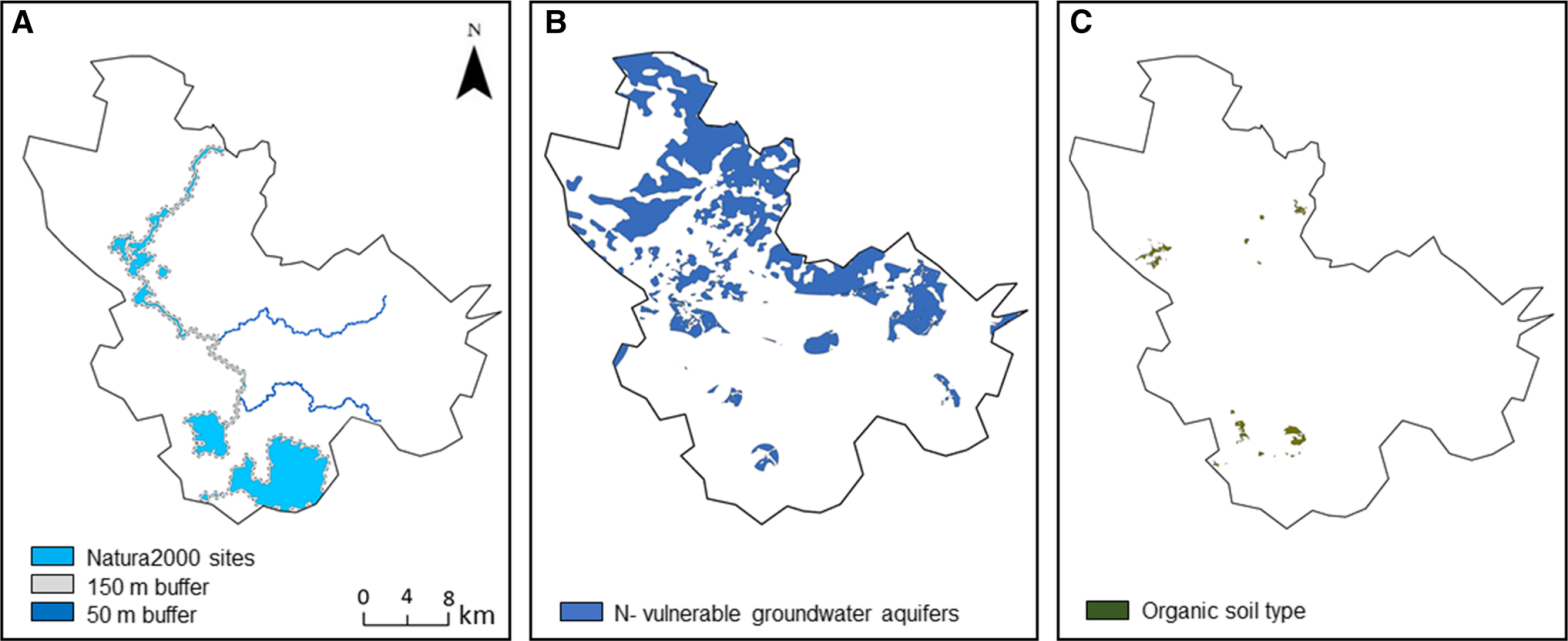
Protected areas within the River Odense catchment: **a** Natura2000 sites with inserted buffer zone, **b** nitrate (N)-vulnerable groundwater aquifers, **c** areas with high organic carbon content in the soils (> 12%)

In strategy (II), two types of protected areas were considered for measure application: (i) Natura2000 sites based on the EU Habitats Directive 1992 and the EU Birds Directive 2009 for nature protection (Fig. 3a) and (ii) nitrate (N)-vulnerable groundwater aquifers based on the EU Nitrate Directive 1991 for groundwater protection (Fig. 3b). The Natura2000 sites include areas covered by the Habitats and Birds directives and RAMSAR, designated to protect specific species and habitats in the EU (Danish Environmental Protection Agency 2019), and within the River Odense catchment these were considered as protected sites located within buffers along watercourses covering an area of 5308 ha including 2296 ha of agricultural lands. The buffer width was defined as a function of the stream width because in countries where river valleys are largely underlain by loose sediments such as Denmark, the width of a river valley generally increases with increasing stream size (Sand-Jensen et al. 2006):

- 50 m buffer for stream width < 2 m and streams of unknown width class and
- 150 m buffer for stream widths between 2 and 12 m.

Groundwater N-vulnerability is defined as the sensitivity of an aquifer to N-contamination (Hansen et al. 2016). Since 1985, both European policies and Danish legislation have aimed to protect groundwater resources from the effect of N-load (Hansen et al. 2016). Approximately 19% of the Danish area has been classified as N-vulnerable groundwater areas (Danish Environmental Portal, 2015), covering an area of 10284 ha including 7380 ha of agricultural lands in the Odense catchment.

For strategy II, grid cells related to agricultural lands within protected areas showing hot-spot areas were first prioritised for selection as target areas for set-aside application until the specified percentages of set-aside (5, 10, 20 and 40%) were achieved. If not achieved, set-aside was applied to the entire protected area and combined with targeting hot-spot areas within the catchment, excluding the protected areas. Explanations of the different approaches for strategy II are given below.

#### Strategy II-1

To prioritise Natura2000 sites for measure application, the required area (ha) for each percentage (5, 10, 20 and 40% equalling 1645, 3290, 6580 and 13160 ha, respectively) of set-aside was compared with the entire agricultural area of the Natura2000 site (2296 ha), and different approaches were applied. For 5% set-aside, only hot-spot areas within the Natura2000 sites were targeted, whilst for 10, 20 and 40%, set-aside was applied first to the entire protected area and next to hot-spot areas within the rest of the catchment.

#### Strategy II-2

To prioritise N-vulnerable groundwater aquifers, the required area for each percentage of set-aside was compared with the entire agricultural area of N-vulnerable aquifers (7380 ha). For 5, 10 and 20% set-aside, only hot-spot areas within the protected area were targeted, whilst for 40% a combination was used, applying set-aside to the entire protected area and targeted set-aside in hot-spot areas within the rest of the catchment.

#### Strategy II-3

Prioritising both Natura2000 sites and N-vulnerable groundwater aquifers, set-aside was performed as described for the II-2 strategy. In this case, the sum of the entire agricultural area of Natura2000 sites and N-vulnerable groundwater aquifers (9676 ha) was compared with the required area for each percentage of set-aside.

### Assessment of spatially targeted strategies

The assessment of spatially targeted strategies included four parts:

1. Assessment of the effects of the targeted strategies on GES implied, firstly, calculation of the different percentages of N-load reduction resulting from each strategy for the period 1990–2009. Secondly, N-load reductions for the period 2010–2014 were calculated by multiplication of the baseline N-load for the period 2010–2014 (694 tons) by the calculated percentages of N-load reduction for the period 1990–2009. Then, the calculated N-loads were compared with the N-load reduction target of 266 tons N yr^−1^ related to the period 2010–2014 for the catchment to be obtained by 2027.
2. Assessment of the effects of the targeted strategies for fulfilment of the climate goals in Denmark, implied assignment of the targeted areas within the border of N-vulnerable groundwater aquifers, Natura2000 sites and the rest of the catchment into the different land use categories of forest, wetlands and set-aside, respectively. The carbon sequestration for potential land use types was calculated using standard values of carbon sequestration specified for each land use type (Table 1) as follows:

**Table 1:** Danish standard values of carbon sequestration specified for different land use types (Eriksen et al. 2014)

Land use	Carbon sequestration [tons CO_2_ equivalents/ha^−1^ yr^−1^]
Set-aside	1.7
Forest	3.8
Wetland	
Mineral soils	0.35
Organic soils	31

$${\text{CS}} = (A_{\text{NO}} \times {\text{CS}}_{\text{P}} ) + (A_{\text{NM}} \times {\text{CS}}_{\text{P}} ) + (A_{\text{G}} \times {\text{CS}}_{\text{P}} ) + \left( {(A_{\text{T}} - (A_{\text{NO}} + A_{\text{NM}} + A_{\text{G}} } \right)) \times {\text{CS}}_{\text{P}} )$$

where CS is the carbon sequestration (tons CO_2_ equiv./ha^−1^ yr^−1^) after considering different land use types, $A_{\text{NO}}$ is the targeted area (ha) within the border of Natura2000 sites containing organic soil (Fig. 3c), ${\text{CS}}_{\text{P}}$ is the Danish standard value of carbon sequestration specified for different land use types, $A_{\text{NM}}$ is the targeted area (ha) within the border of Natura2000 sites containing mineral soil, $A_{\text{G}}$ is the targeted area (ha) within the border of N-vulnerable groundwater aquifers, and $A_{\text{T}}$ is the total targeted area (ha) within the rest of the catchment. Further, to compare the effectiveness of scenarios in terms of carbon sequestration, the result of each scenario was divided by the total required reduction in the catchment of 37000 tons CO_2_ equivalents.

3. Assessment of the effects of the targeted strategies on nature protection and groundwater quality was performed for both protected areas—Natura2000 sites and N-vulnerable groundwater aquifers—by dividing the set-aside areas resulting from each strategy located within the border of each protected area by the total area of each protected area.
4. Cost assessment of the multi-functional benefits of targeted strategies was performed by dividing the total cost (i.e. welfare cost of agricultural lands being out of production) of each strategy presented in Fig. 2 by the total multi-functional benefits (i.e. maximum score of 400 points) gained from each strategy. Maximum score benefit is the total sum of benefits related to GES (maximum 100 for obtaining the N-load reduction target for the catchment), restoration of groundwater chemical quality (maximum 100 for covering the entire area above N-vulnerable groundwater aquifers with set-aside), protection of nature (maximum 100 for covering all Natura2000 area with set-aside) and fulfilment of formerly set climate goals in Denmark (maximum 100 for obtaining Danish climate goals). The total cost of each strategy was calculated using Danish standard values for the welfare cost (i.e. the total cost to society that is not limited only to farmers) of set-aside (€ ha^−1^ yr^−1^) (Table 2). This was performed by, firstly, identifying the area and location of targeted set-aside for each strategy and, secondly, by considering farm and soil types, each area being multiplied by the related welfare cost value. The soil type data in this study were derived from the root zone database developed for Denmark at Aarhus University (Børgesen et al. 2013) (Fig. 1b), including 51% clayey soil and 49% sandy soil. Information about crops and farmland boundaries was available for 2011 at field scale from the General Farm Register (GLR in Danish), and this was combined with information on the use of fertiliser and manure from the Danish AgriFish Agency (Dalgaard et al. 2002). Considering the farm types in Table 2, the Odense catchment holds 11%, 75%, 7% and 7% of farm numbers 1, 2, 3 and 4, respectively.

**Table 2:** Danish standard values for the welfare cost of set-aside (€/ha^−1^ yr^−1^) specified for available farm types based on different soil types and application of manure (Eriksen et al. 2014)

Farm No.	Farm type	Welfare cost of set-aside [€ ha^−1^ yr^−1^]	
		Sandy soil	Clayey soil
1	Plant/pig farm without manure	292	964
2	Plant/pig farm with manure	489	1188
3	Cattle farm without manure	223	471
4	Cattle farm with manure	534	745

## Results

### Effectiveness of targeted set-aside

#### Restoration of ecology in estuary (strategy I)

**Fig. 4:**
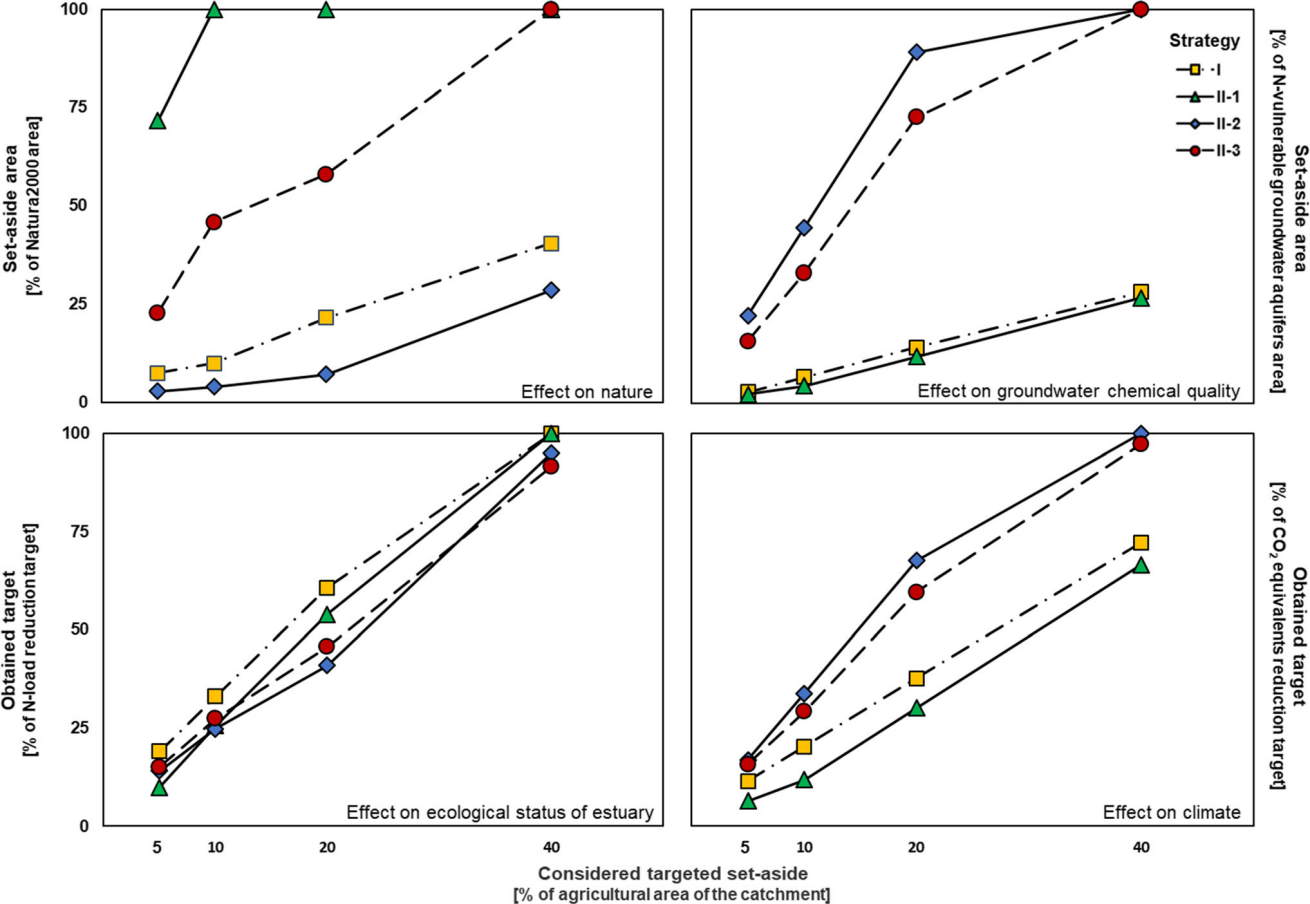
Effect of spatially targeted strategies on nature (% of targeted set-aside located within the Natura2000 border), groundwater chemical quality (% of targeted set-aside located within the border of N-vulnerable groundwater aquifers), ecological status of estuary (% of N-load reduction target) and climate (% of Danish climate goals for carbon sequestration). Strategies for set-aside application are I: set-aside in high N-load areas within the entire catchment area, II-1: set-aside prioritising Natura2000, II-2: set-aside prioritising N-vulnerable groundwater aquifers, II-3: set-aside prioritising both Natura2000 and N-vulnerable groundwater aquifers. Considered targeted set-aside percentages of the agricultural area of the River Odense catchment are 5%, 10%, 20% and 40%

5% set-aside resulted in 19% fulfilment for GES (% of N-load reduction target for the catchment), whereas fulfilment for nature (% of targeted set-aside located within the Natura2000 border), groundwater (% of targeted set-aside located within the border of N-vulnerable groundwater aquifers) and climate (% of Danish climate goals) amounted to 8%, 3% and 11%, respectively (Fig. 4). 40% set-aside resulted in 100%, 40%, 72% and 28% fulfilment for GES, nature, climate and groundwater, respectively. The results of using 10% and 20% targeted set-aside showed the same trends as the 5% and 40% set-aside.

#### Protection of nature (strategy II-1)

5% set-aside resulted in 72%, 2%, 6% and 10% fulfilment for nature, groundwater, climate and GES, respectively (Fig. 4). 40% set-aside resulted in 100% fulfilment for both nature and GES and 27% and 67%, respectively, for groundwater and climate. 10% and 20% set-aside revealed the same trends as 5% and 40% set-aside.

#### Protection of N-vulnerable groundwater aquifers (strategy II-2)

5% set-aside resulted in 22% fulfilment for groundwater and 3%, 17% and 14% for nature, climate and GES, respectively (Fig. 4). 40% set-aside yielded 100% fulfilment for both climate and groundwater and 29% and 95% for nature and ecology, respectively. 10% and 20% set-aside demonstrated the same trends as 5% set-aside.

#### Protection of both nature and N-vulnerable groundwater aquifers (strategy II-3)

5% set-aside gave 23%, 16%, 16% and 15% fulfilment and 10% set-aside 46%, 33%, 29% and 27% fulfilment for nature, groundwater, climate and GES, respectively (Fig. 4). 20% set-aside resulted in 58%, 73%, 60% and 46% fulfilment and 40% set-aside in 100%, 100%, 97% and 92% fulfilment for nature, groundwater, climate and GES, respectively.

### Cost–benefit of targeted set-aside

**Fig. 5:**
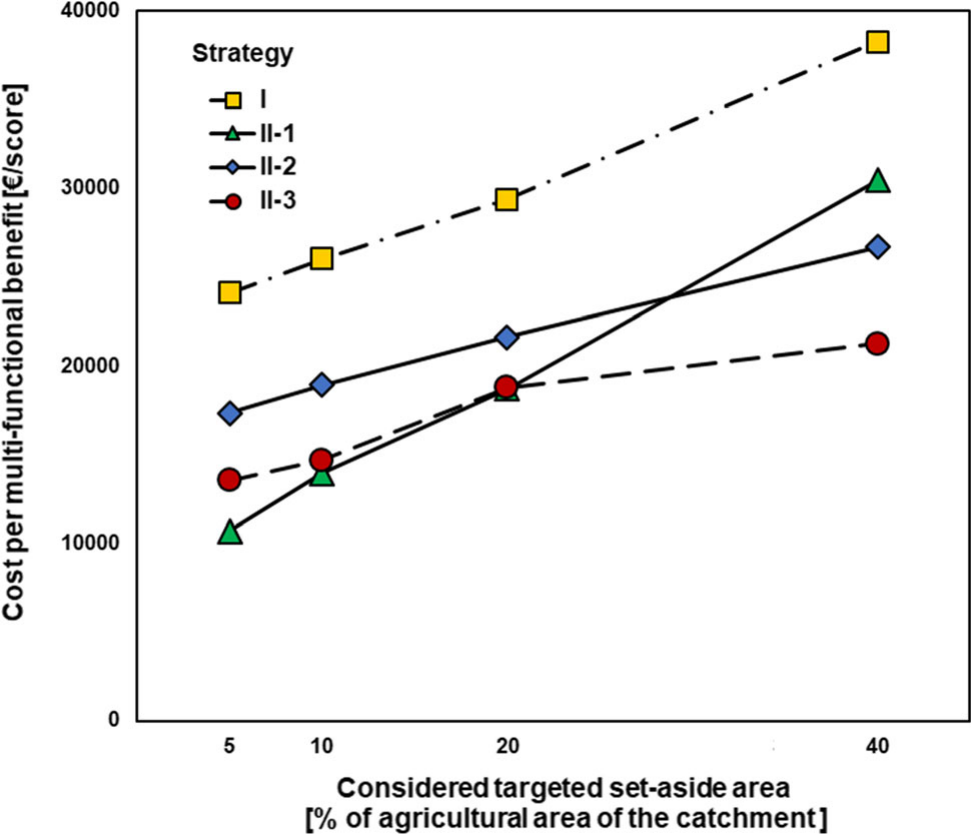
Cost per multi-functional benefit (**€**/score) considering different spatially targeted strategies and four different percentages (5, 10, 20 and 40%) for set-aside application. Strategies for set-aside application are I: set-aside in high N-load areas within the entire catchment area, II-1: set-aside prioritising Natura2000, II-2: set-aside prioritising N-vulnerable groundwater aquifers, II-3: set-aside prioritising both Natura2000 and N-vulnerable groundwater aquifers

The costs of targeting set-aside to gain multi-functional benefits are presented in Fig. 5. Maximum cost per achieved multi-functional score benefit was found for Strategy I targeting hot-spot areas within the entire catchment area, whilst strategy II-1 targeting Nature2000 areas involved the minimum cost for 5, 10 and 20%, but not 40%, set-aside. For strategy II-2 with prioritised targeting set-aside in N-vulnerable groundwater aquifers, the cost per multi-functional score benefit was generally lower than for strategy I but higher than for strategy II-1 and II-3. For strategy II-3, the cost per multi-functional score benefit was overall lowest for 20% and 40% set-aside.

## Discussion

### Potential multi-functional benefits of targeted set-aside

Our use of protective set-aside buffer zones around the Natura 2000 areas in the River Odense at Kratholm sub-catchment follows up on the requirements for all EU Member States to improve the ecological coherence of Natura2000 sites (Oenema et al. 2011). Use of set-aside in combination of afforestation will assist in developing new features of the landscape that imply restrictions on the current agricultural activities within and around the Natura 2000 areas. Forested buffer zones around Natura 2000 areas in the River Odense at Kratholm catchment will safeguard biodiversity by lowering local NO_3_^−^ emissions to water as well as NH_3_ and NO_x_ emissions to air (Bastrup-Birk and Gundersen 2004; Hertel et al. 2011).

The conservation of groundwater protected nitrate vulnerable areas in the Kratholm catchment using set-aside aim to protect water resources on a larger scale in groundwater recharge areas (Oenema et al. 2011). Our use of afforestation in these areas is one of the main conservation methods that will help to improve and preserve upper and deeper groundwater quality on a longer time scale for threats from pesticide and nitrate pollution (Bastrup-Birk and Gundersen 2004). Such a set-aside with afforestation will help to fulfil the EU Nitrates Directive, Water Framework Directive and Groundwater Directive goals of reversing pollution trends and prevent and limit inputs of pollutants into groundwater (Oenema et al. 2011).

Further, the use of set-aside in the targeted areas is intended to support the change from use of fossil fuels towards renewable resources ‘green shift’ that will increase the demand for biomass production in the Nordic countries (Marttila et al. 2020). Afforestation in nitrate vulnerable groundwater areas, restoration of wetlands in the buffer zones around Natura2000 areas with production of straw (e.g. Paludicultures) and the set-aside in agricultural areas that can be used for harvesting of straw from grass and herbs will assist in such a future societal transformation towards a more circular bio-based economy.

Our results show that targeted strategies for restoration of the ecology in Odense estuary (strategy I) adopted in hot-spot areas leads to higher N-load reduction compared to other targeted strategies. The extent of potential N-load reduction to the estuary is influenced by application of other strategies such as prioritising set-aside application for protection of Natura2000 areas or N-vulnerable groundwater aquifers within the River Odense catchment.

Fulfilment of the target N-load for the 40% set-aside strategies comes at the cost of diminishing returns, i.e. the high N-load reduction requires that set-aside is applied to a large quantity of arable lands where the obtained reduction per ha is small (equivalent to diminishing returns) (Hashemi et al. 2018a). In addition, the catchment includes non-agricultural areas contributing to the N-load, so the relative proportion of the N-load from agricultural areas that needs to be reduced is equal to the percentage of the target load reduction, and since set-aside does not eliminate leaching, the targeting of set-aside actually enhances efficiency. The findings of our study confirm the results of an investigation by Hashemi et al. (2018a), who applied cover crops and set-aside to decrease N-leaching, and this more than doubled the required area to obtain a doubled N-load reduction target. Also, they included a scenario for N-leaching relocation based on N-reduction considering spatial constraints (e.g. soil type and farm boundary), whereas our study considered protected areas; therefore, our two studies are not directly comparable. However, Hashemi et al. (2018a) found a lower need for set-aside than in our study, suggesting that a combination of targeted mitigation measures and N-leaching relocation may be more cost-efficient than merely targeting mitigation measures at different parts of the landscape.

Considering all four percentages of set-aside, the maximum effectiveness for both groundwater protection and climate was obtained by prioritising the N-vulnerable groundwater aquifers (strategy II-2), whilst strategies II-1 and I had the maximum effect on, respectively, nature protection and GES. Indeed, the potential benefit of applying set-aside to N-vulnerable groundwater aquifers to decrease N-load was affected by the size of the area (ha) and by the N-reduction in the groundwater, whilst application of set-aside to Natura2000 sites to decrease the N-load affected a limited targeting area but entailed benefits for nature protection. The potential of set-aside application to both protected areas for carbon sequestration is also affected by the size of the area (ha) and by the specified standard value of carbon sequestration for different land use types.

Rakovic et al. (2020) presents a method to downscale and extend the global Shared Socioeconomic Pathways (SSPs) into a set of storylines focused on the Nordic land-based bioeconomy—the Nordic Bioeconomy Pathways (NBPs). The narrative storyline, NBP1—Sustainability first, Closing the Loops, and some of its associated land management attributes that are meant to translate the qualitative NBP storyline into the quantitative data needed for alternative future socioeconomic and water quality scenarios at catchment scale, is closely linked to our study of the multi-functional benefits of targeted land use changes in the River Odense catchment. The three agricultural and forestry attributes that we also explored were: (i) implementation of mitigation measures where land is taken out of production—set aside; (ii) catchment management strategy and (iii) land cover.

### Potential cost of multi-functional benefits of targeted set-aside

Our results on the major co-benefits of targeted strategies were related to the economic value of set-aside to elucidate the costs incurred to obtain various benefits and to investigate different strategies based on their cost effectiveness. This allows decision makers to choose new solutions for meeting different societal objectives based on a targeted and cost-minimising spatial location of measures.

Figure 5 shows that prioritisation of Natura2000 areas for targeted application (strategy II-1) of set-aside in 5, 10 and 20% of agricultural lands led to the highest multi-functional score benefit and thereby the lowest cost compared with the other strategies. This is simply because targeting set-aside to hot-spot areas within the Natura2000 sites resulted in both N-load reduction and nature protection. Furthermore, Natura2000 areas contain organic soils to which wetland standard values of carbon sequestration are applied that increased the value of carbon sequestration. However, when considering N-vulnerable groundwater aquifers the observed effects are greatly influenced by the size of the prioritised areas (strategy II-2) and when considering both N-vulnerable groundwater aquifers and Natura2000 sites for set-aside application (strategy II-3), the cost per multi-functional score benefit was lower for the 40% set-aside compared to strategy II-1. In this study, considering 5% set-aside as a realistic target area, the optimal strategy is II-1 (protection of nature) and the same is true for the other set-aside target areas, except for the 40% target area where the mixed II-3 strategy is the optimal one.

Our study shows that if set-aside is applied in both hot-spot and protected areas, the potential multi-functional benefits of targeted strategies will increase at low cost in the Odense catchment. However, the location of the Natura2000 sites and their vicinity to the rivers are very important to obtain co-benefits of targeted set-aside such as restoration of riparian wetlands (Audet et al. 2020). This important finding needs to be corroborated by studies in other catchments varying in geology, land use and protected areas.

### Evaluation of methodology

Some issues related to designing the strategies may limit the potential co-benefits of them, being related to the way that the land use change is implemented. Our study assumed that landscape management could be adjusted at 200 m scale, but the scale of adjusting land management within a farm may differ depending on field size and associated demands for mechanisation (Hashemi et al. 2018a). In practice, the use of targeted land use change may also prove difficult because some farmers would face significantly stricter requirements to set-aside areas than others, and especially farmers with a high livestock ratio might find it impossible to fulfil the general livestock harmony rules of the EU Nitrate Directive, amounting to 1.7 Livestock Units ha^−1^. Thus, the scientific underpinning for differentiated treatment of farmers may become an issue together with potential interference with private property rights. Use of targeted green shift (set-aside), therefore, requires careful implementation and local knowledge when identifying the relevant sites for measure application. Such issues can to some extent be managed through subsidies and public support. Protected areas are also targeted to specific locations within the catchment. It could be argued that it would be more appropriate to use a more spatially differentiated approach by applying other measures to decrease N-leaching and increase N-reduction. In this way, less agricultural lands would be out of production and other benefits such as biomass production would be considered.

Furthermore, the 12 kg N ha^−1^ yr^−1^ used in our analysis for set-aside is considered as a baseline N-leaching value and as forests mature and the C/N ratio in the agricultural soil content increases, the N-leaching value will decrease to 2 kg N ha^−1^ yr^−1^, and the effect of the strategies will become stronger than the estimated values.

Finally, uncertainties of input maps (N- reduction and N-leaching) used in N-load calculations could be a major source of uncertainty that will propagate to the estimated multi-functional benefits. In our study, it was assumed that the N-reduction and N-leaching maps have no uncertainties, but in reality their uncertainty will affect the outcome of different spatially targeted strategies. However, based on the study by Hashemi et al. (2018b) on reducing the uncertainty of estimated N-load reductions through spatially targeting of set-aside by considering high-resolution groundwater N-reduction maps, it could be possible to reduce the uncertainty of estimated results of spatially targeted measures.

## Conclusions

Our work demonstrates how geographically targeted set-aside in hot-spot and protected areas within a catchment can reduce the N-load to vulnerable ecosystems such as estuaries and at the same time promote a range of benefits such as protection of groundwater and nature and climate change mitigation. Our results show that lowering the cost of obtaining multi-functional benefits requires a targeting of set-aside areas in catchments to designated protected areas where multiple effects are more than when focusing solely on hot-spot N-loss areas. This implies that catchment screening of both hot-spot nutrient load areas and other areas providing important benefits is needed before application of land use changes to maximise potential multi-functional benefits at the lowest cost.
